# Supplementary material for: Oral Microbiota Linking Associations of Dietary Factors with Recurrent Oral Ulcer
Source: Nutrients. 2024 May 17;16(10):1519. doi: 10.3390/nu16101519 (PMC11124033; doi:10.3390/nu16101519)
Supplement: Supplementary file 1 [file nutrients-16-01519-s001.zip › nutrients-2989937-supplementary.pdf]

Supplementary Materials

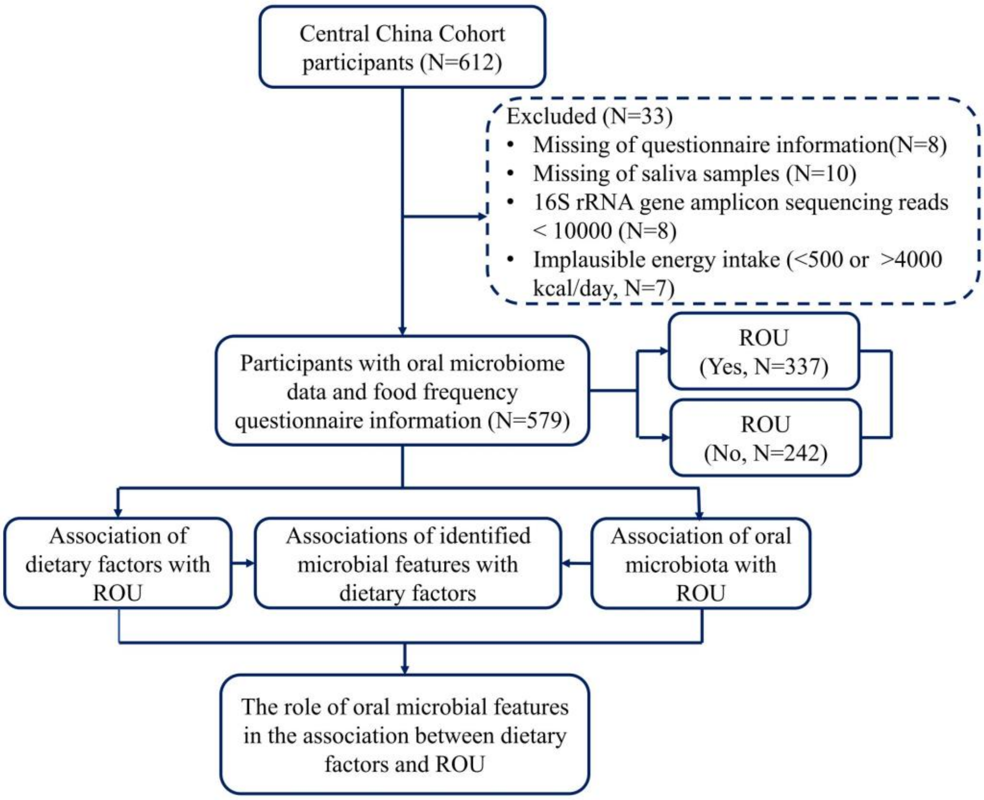

Figure S1. Flow-chart of study participants included in this study from Central China Cohort. *ROU* recurrent oral ulcer, *16S rRNA* 16S ribosomal RNA.

Dietary factors	OR (95% CI)	*P* value	*P* trend
PDI			0.142
T2 vs T1	0.68(0.32−1.48)	0.333	
T3 vs T1	0.55(0.23−1.31)	0.176	
hPDI			0.868
T2 vs T1	1.17(0.51−2.69)	0.715	
T3 vs T1	0.91(0.37−2.22)	0.830	
uPDI			0.334
T2 vs T1	0.48(0.22−1.08)	0.078	
T3 vs T1	0.63(0.25−1.56)	0.314	
Refined grains			0.563
T2 vs T1	1.64(0.68−3.96)	0.274	
T3 vs T1	0.99(0.41−2.35)	0.973	
Whole grains			0.166
T2 vs T1	0.65(0.14−2.98)	0.583	
T3 vs T1	0.63(0.30−1.32)	0.218	
Legumes			0.948
T2 vs T1	2.18(0.95−4.99)	0.065	
T3 vs T1	1.34(0.56−3.18)	0.510	
Vegetables			0.719
T2 vs T1	1.07(0.48−2.37)	0.876	
T3 vs T1	0.88(0.36−2.13)	0.770	
Mushroom			0.477
T2 vs T1	1.66(0.74−3.71)	0.217	
T3 vs T1	1.73(0.70−4.31)	0.236	
Fruits			0.729
T2 vs T1	1.32(0.60−2.91)	0.488	
T3 vs T1	1.13(0.48−2.65)	0.777	
Nuts			0.980
T2 vs T1	0.72(0.32−1.66)	0.443	
T3 vs T1	0.87(0.39−1.95)	0.733	
Red meat			0.010
T2 vs T1	1.28(0.51−3.20)	0.596	
T3 vs T1	3.01(1.15−7.85)	0.024	
Processed meat			0.398
T2 vs T1	1.03(0.44−2.41)	0.945	
T3 vs T1	1.96(0.66−5.81)	0.226	
Poultry			0.087
T2 vs T1	1.41(0.61−3.27)	0.416	
T3 vs T1	2.45(0.93−6.47)	0.071	
Fish			0.686
T2 vs T1	1.30(0.56−3.03)	0.543	
T3 vs T1	1.22(0.54−2.74)	0.637	
Dairy			0.057
T2 vs T1	1.15(0.56−2.38)	0.705	
T3 vs T1	2.84(0.97−8.27)	0.056	
Eggs			0.635
T2 vs T1	0.63(0.29−1.37)	0.240	
T3 vs T1	1.23(0.47−3.22)	0.675	

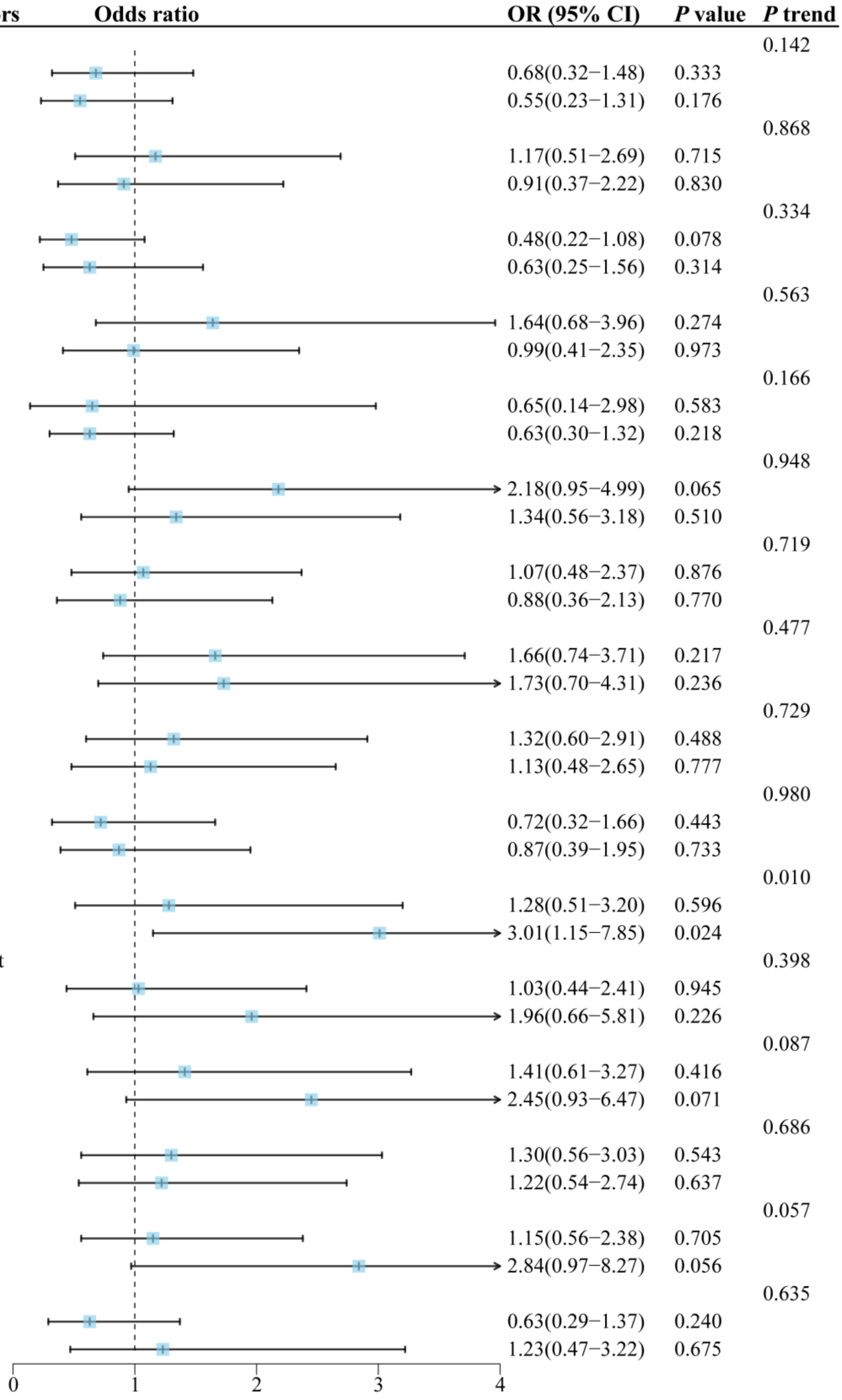

Figure S2. Associations of dietary factors with recurrent oral ulcer in males. Odds ratios were derived from logistic regression models for tertiles 2 (T2) and tertiles 3 (T3) of included dietary factors using tertiles 1 (T1) as the reference group. Covariates included age, current smoking status, physical activity, total energy intake, BMI, and common chronic diseases. *PDI* plant-based diet index, *hPDI* healthful plant-based diet index, *uPDI* unhealthful plant-based diet index.

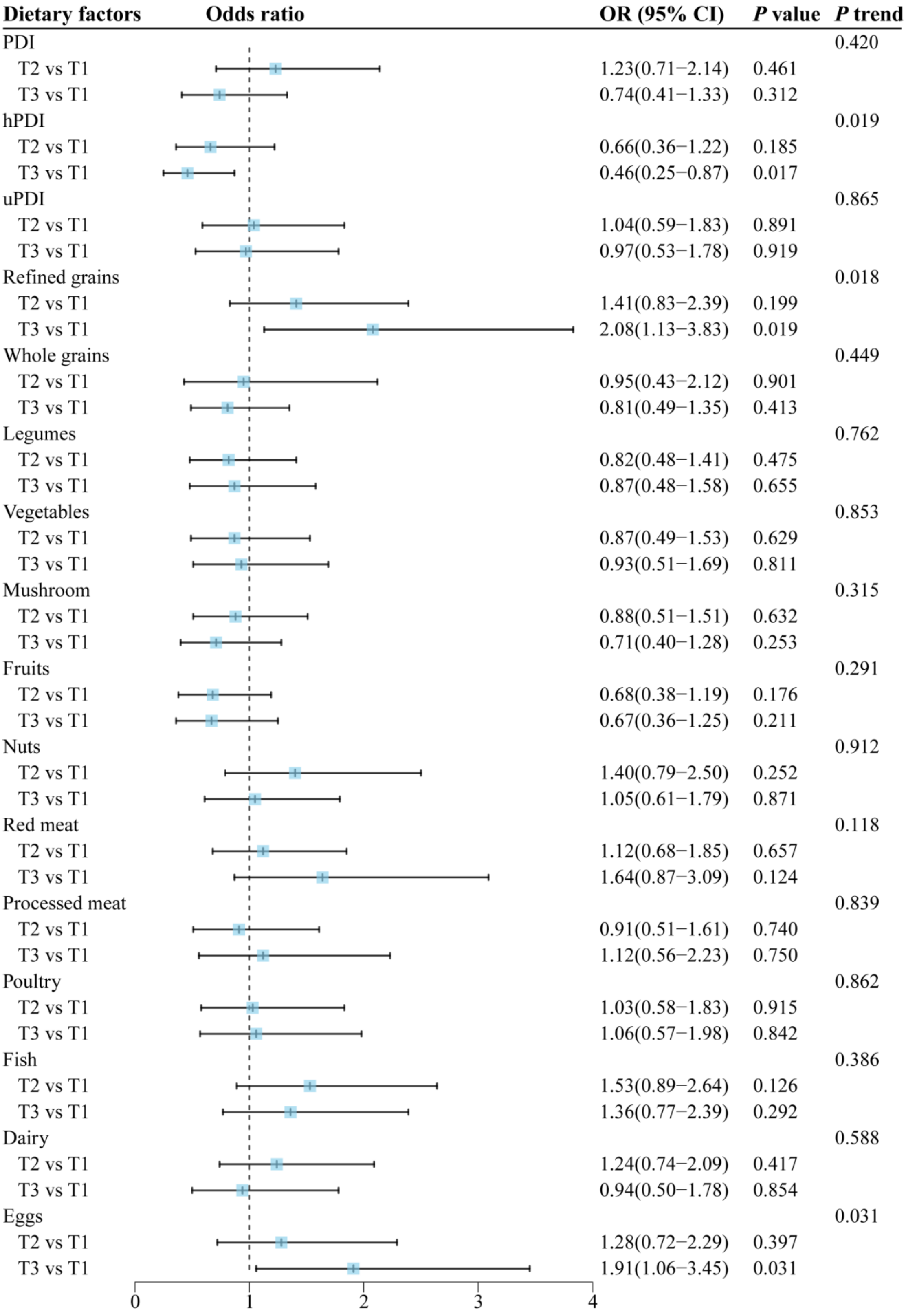

Dietary factors	OR (95% CI)	*P* value	*P* trend
PDI			0.420
T2 vs T1	1.23(0.71−2.14)	0.461	
T3 vs T1	0.74(0.41−1.33)	0.312	
hPDI			0.019
T2 vs T1	0.66(0.36−1.22)	0.185	
T3 vs T1	0.46(0.25−0.87)	0.017	
uPDI			0.865
T2 vs T1	1.04(0.59−1.83)	0.891	
T3 vs T1	0.97(0.53−1.78)	0.919	
Refined grains			0.018
T2 vs T1	1.41(0.83−2.39)	0.199	
T3 vs T1	2.08(1.13−3.83)	0.019	
Whole grains			0.449
T2 vs T1	0.95(0.43−2.12)	0.901	
T3 vs T1	0.81(0.49−1.35)	0.413	
Legumes			0.762
T2 vs T1	0.82(0.48−1.41)	0.475	
T3 vs T1	0.87(0.48−1.58)	0.655	
Vegetables			0.853
T2 vs T1	0.87(0.49−1.53)	0.629	
T3 vs T1	0.93(0.51−1.69)	0.811	
Mushroom			0.315
T2 vs T1	0.88(0.51−1.51)	0.632	
T3 vs T1	0.71(0.40−1.28)	0.253	
Fruits			0.291
T2 vs T1	0.68(0.38−1.19)	0.176	
T3 vs T1	0.67(0.36−1.25)	0.211	
Nuts			0.912
T2 vs T1	1.40(0.79−2.50)	0.252	
T3 vs T1	1.05(0.61−1.79)	0.871	
Red meat			0.118
T2 vs T1	1.12(0.68−1.85)	0.657	
T3 vs T1	1.64(0.87−3.09)	0.124	
Processed meat			0.839
T2 vs T1	0.91(0.51−1.61)	0.740	
T3 vs T1	1.12(0.56−2.23)	0.750	
Poultry			0.862
T2 vs T1	1.03(0.58−1.83)	0.915	
T3 vs T1	1.06(0.57−1.98)	0.842	
Fish			0.386
T2 vs T1	1.53(0.89−2.64)	0.126	
T3 vs T1	1.36(0.77−2.39)	0.292	
Dairy			0.588
T2 vs T1	1.24(0.74−2.09)	0.417	
T3 vs T1	0.94(0.50−1.78)	0.854	
Eggs			0.031
T2 vs T1	1.28(0.72−2.29)	0.397	
T3 vs T1	1.91(1.06−3.45)	0.031	

Figure S3. Associations of dietary factors with recurrent oral ulcer in females. Odds ratios were derived from logistic regression models for tertiles 2 (T2) and tertiles 3 (T3) of included dietary factors using tertiles 1 (T1) as the reference group. Covariates included age, current smoking status, physical activity, total energy intake, BMI, and common chronic diseases. *PDI* plant-based diet index, *hPDI* healthful plant-based diet index, *uPDI* unhealthful plant-based diet index.

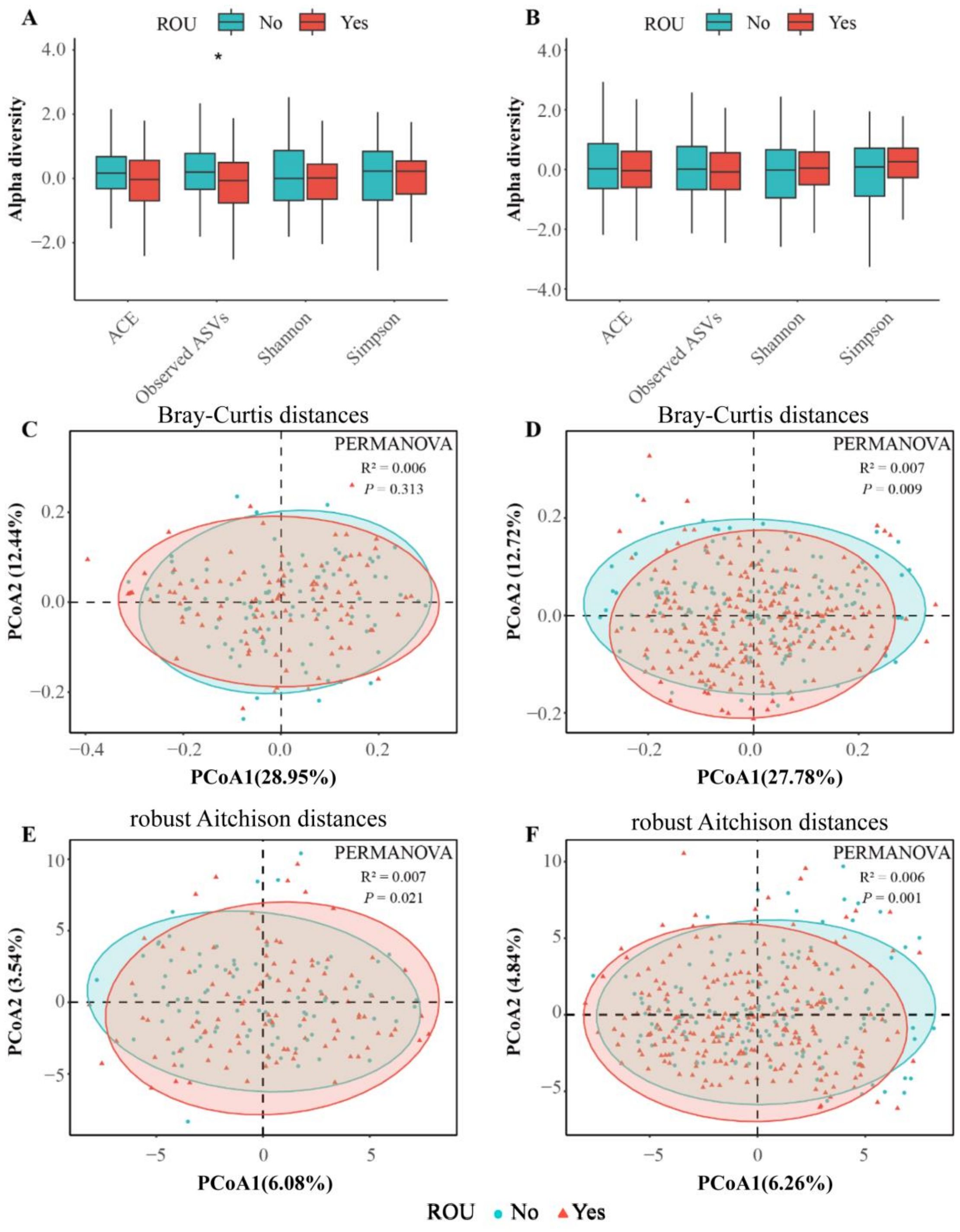

Figure S4. Diversity estimates and relative abundance of oral microbiota associated with ROU in males (A, C, E) and females (B, D, F). (A, B) Comparisons of ACE, Observed ASVs, Shannon and Simpson indices in the oral microbiota between participants with or without ROU in males (A) and females (B). * $p < 0.05$. All four indices were z-score standardized for visualization. (C, D) PCoA based on the Bray-Curtis distances of the oral microbial communities between participants with or without ROU in males (C) and females (D). (E, F) PCoA based on the robust Aitchison distances of the oral microbial communities between participants with or without ROU in males (E) and females (F). *ROU* recurrent oral ulcer.

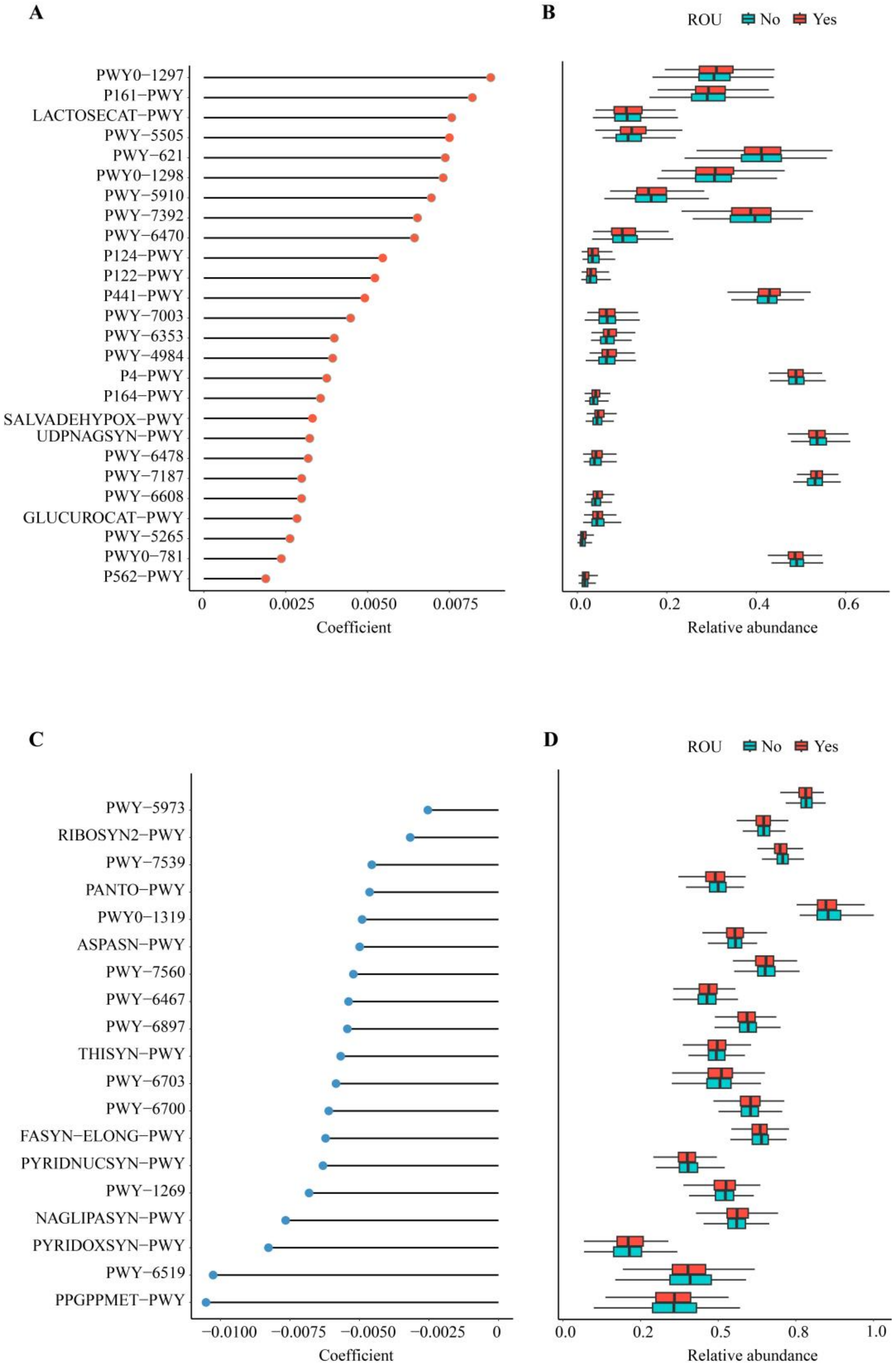

Figure S5. The relative abundance difference of ulcer-associated functional pathways. (A, C) The lollipop charts show the coefficients of pathways derived from MaAsLin analysis with *p*-FDR < 0.20. (B, D) The box plots show the relative abundances of differentially abundant pathways between participants with or without ROU. *ROU* recurrent oral ulcer.

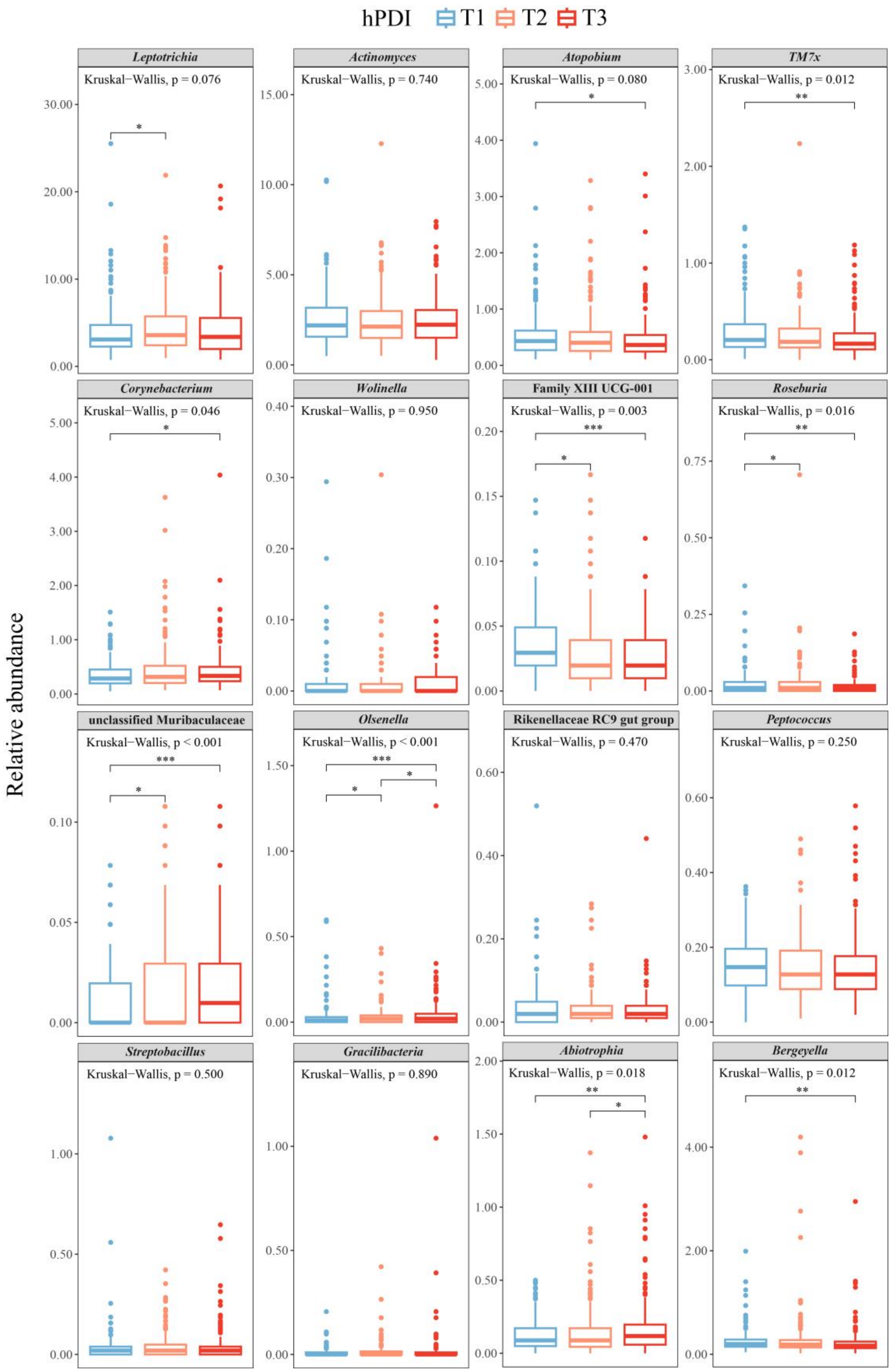

Figure S6. The relative abundance difference of 16 identified genera according to tertiles of healthful plant-based diet index. * $p < 0.05$, ** $p < 0.01$, *** $p < 0.001$. *hPDI* healthful plant-based diet index.

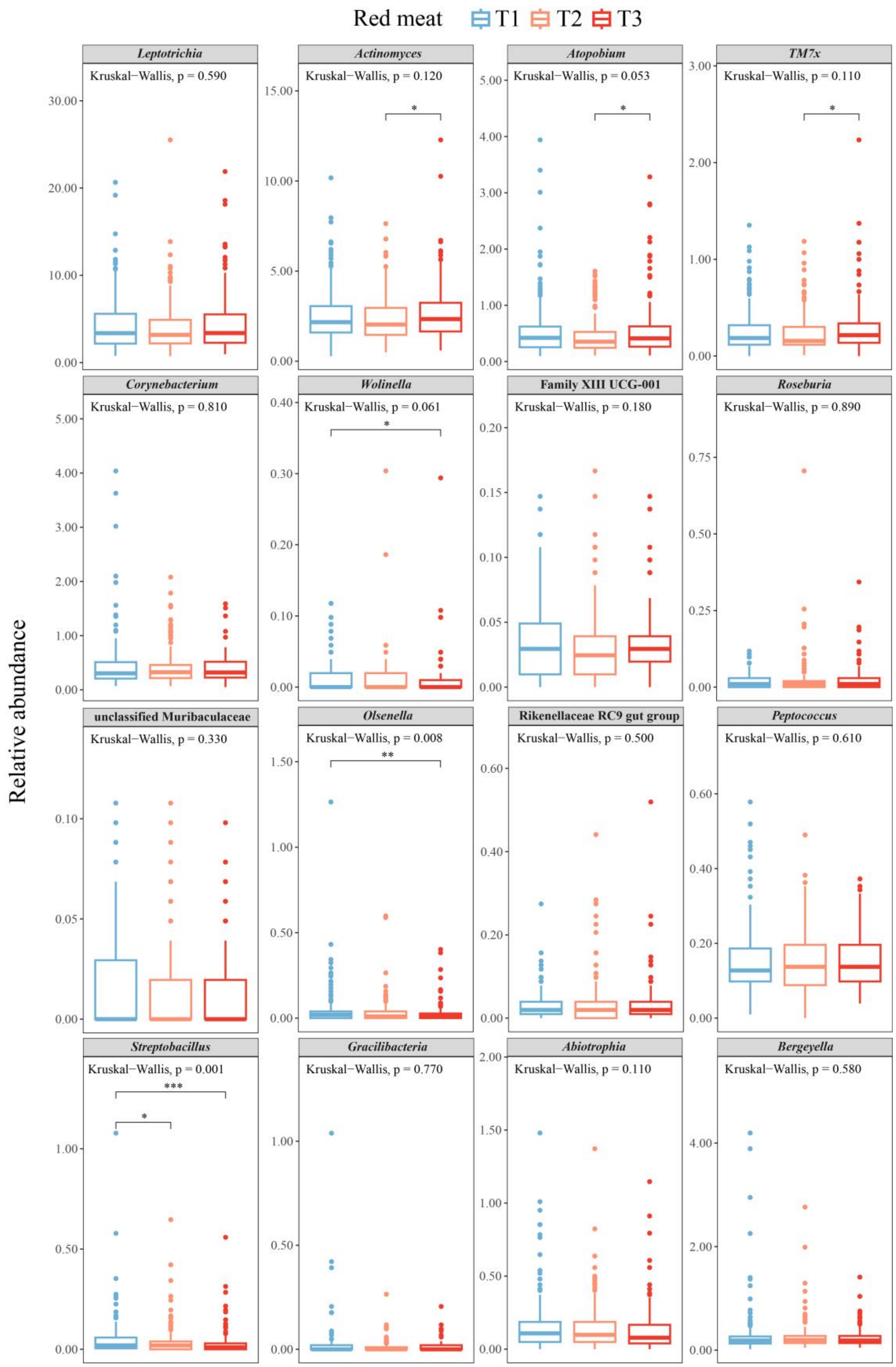

Figure S7. The relative abundance difference of 16 identified genera according to tertiles of red meat intake. * $p < 0.05$, ** $p < 0.01$, *** $p < 0.001$.

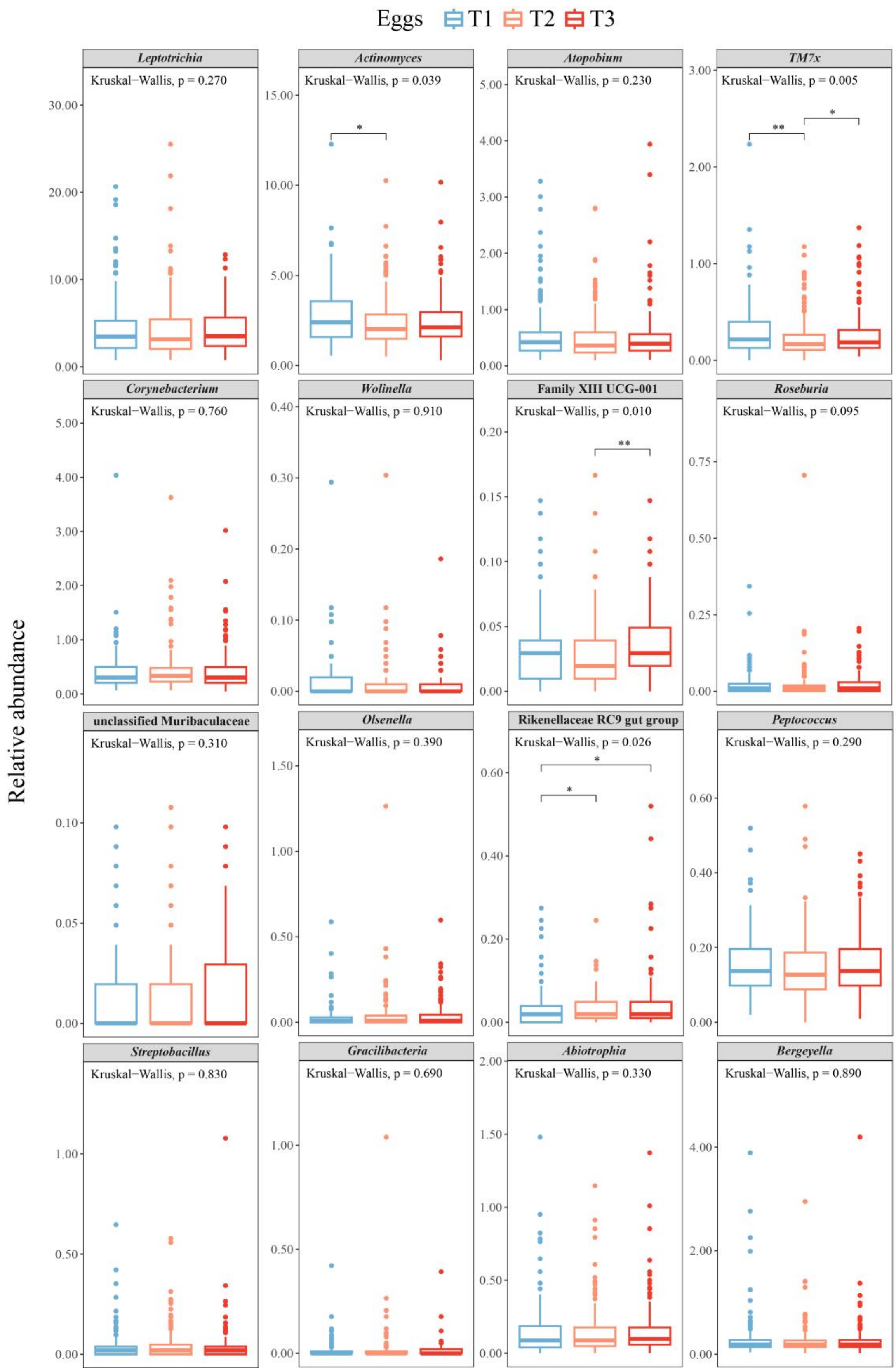

Figure S8. The relative abundance difference of 16 identified genera according to tertiles of egg intake. * $p < 0.05$, ** $p < 0.01$.

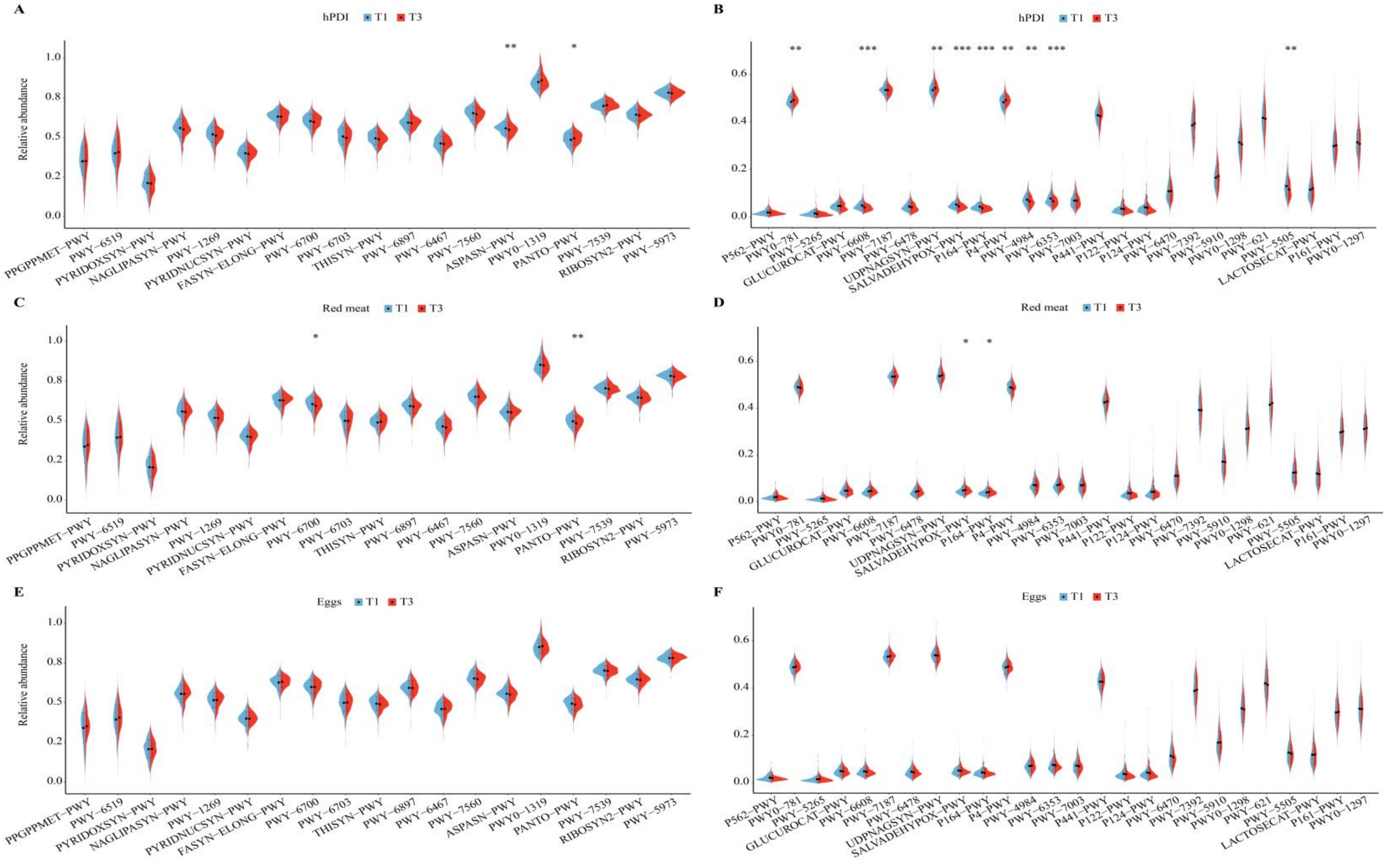

Figure S9. The relative abundance difference of ulcer-associated functional pathways among specific dietary factors. (A, B, C, D, E, F) The relative abundance difference of 45 pathways between T1 and T3 groups of hPDI, red meat and egg intakes. * $p < 0.05$, ** $p < 0.01$, *** $p < 0.001$. *hPDI* healthful plant-based diet index.

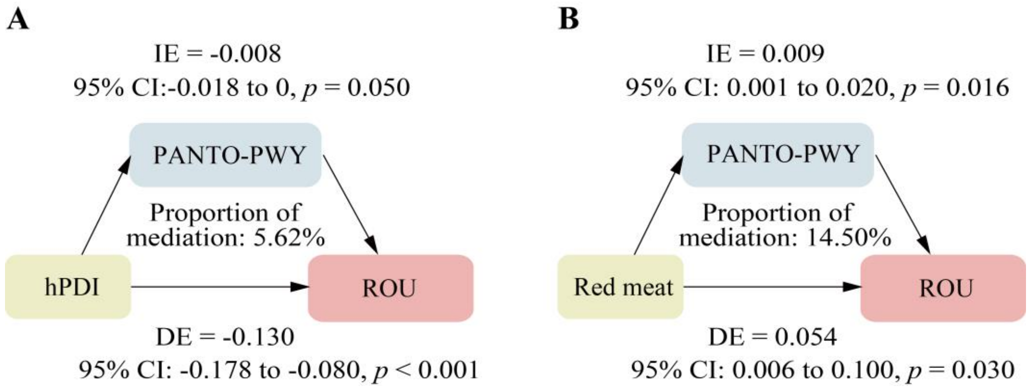

Figure S10. Associations mediation effect of functional pathways among the association between specific dietary factors and recurrent oral ulcer. The direct effect (DE) refers to the effect of dietary factors on ROU when pathways were held at a level. The indirect effect (IE) refers to the impact of dietary factors on ROU through pathways. Proportion of mediation represents the percentage of the association between dietary factors and ROU that is explained by pathways. *ROU* recurrent oral ulcer, *hPDI* healthful plant-based diet index, *IE* indirect effect, *DE* direct effect.

Table S1. Food groups, daily intakes and score assignments used to calculate plant-based diet indices*.

Food Group	Food item(s)	Daily intake (g/day)	Score assignment (18~90)		
			hPDI	PDI	uPDI
Whole grains	Coarse grains	<14.96	1	1	5
		14.96-49.08	2	2	4
		49.08-70.77	3	3	3
		70.77-102.04	4	4	2
		>102.04	5	5	1
Fruits	Oranges, pomelos, tangerines, apple, pear, peach, plum, loquat, jujube, apricot, strawberries, blueberries, grapes, watermelon, melon, and other fruits (pineapple, lychee, mango, banana, etc.)	<77.85	1	1	5
		77.85-145.82	2	2	4
		145.82-219.64	3	3	3
		219.64-294.19	4	4	2
		>294.19	5	5	1
Vegetables	Greens (spinach, cabbage, lettuce, etc.), cauliflower, broccoli, kale, carrots, tomatoes, squash, lotus root, yam, white radish, asparagus, eggplant, fresh green/red pepper, cucumber, white gourd, loofah, zucchini, bitter gourd, celery, cabbage moss, onion, scallion, garlic, leek, etc.	<246.07	1	1	5
		246.07-309.21	2	2	4
		309.21-388.59	3	3	3
		388.59-475.71	4	4	2
		>475.71	5	5	1
Nuts	Peanuts, sunflower seeds, chestnuts, walnuts, etc.	<1.95	1	1	5
		1.95-3.12	2	2	4
		3.12-4.47	3	3	3

		4.47-12.96	4	4	2
		>12.96	5	5	1
Legumes	Soybean milk, fresh tofu, dried bean curd and all kinds of bean products, soybeans, green beans, black beans, edamame, lentils, cowpeas, yellow bean sprouts, mung bean sprouts, etc.	<10.18	1	1	5
		10.18-16.47	2	2	4
		16.47-24.15	3	3	3
		24.15-42.46	4	4	2
		>42.26	5	5	1
Vegetable oils	Soy bean oil, peanut oil, olive oil, and seed oil	<0.98	1	1	5
		0.98-1.02	2	2	4
		1.02-1.06	3	3	3
		1.06-3.38	4	4	2
		>3.38	5	5	1
Tea and coffee	Green tea, black tea, oolong tea, flower tea, Pu-erh tea, and coffee	$<0.71\times10^{-4}$	1	1	5
		0.71×10^{-4}-1.25×10^{-4}	2	2	4
		1.25×10^{-4}-2.54×10^{-4}	3	3	3
		2.54×10^{-4}-1.90×10^{-3}	4	4	2
		$>1.90\times10^{-3}$	5	5	1
Fruit juices	100% pure juice and other juices	<34.92	5	1	1
		34.92-37.66	4	2	2
		37.66-41.10	3	3	3
		41.10-57.34	2	4	4
		>57.34	1	5	5
Refined grains	Rice, noodles and steamed bread	<114.96	5	1	1
		114.96-147.72	4	2	2
		147.72-185.27	3	3	3
		185.27-232.15	2	4	4

		>232.15	1	5	5
Potatoes	Potato, sweet potato, etc.	<13.72	5	1	1
		13.72-43.44	4	2	2
		43.44-60.91	3	3	3
		60.91-76.33	2	4	4
		>76.33	1	5	5
Sugar sweetened beverages	Coke and other carbonated drinks, and artificial sweetener or low sugar drinks	<46.51	5	1	1
		46.51-49.38	4	2	2
		49.38-54.03	3	3	3
		54.03-90.59	2	4	4
		>90.59	1	5	5
Sweets and desserts	Pastries, biscuits, cakes, candy preserves, ice cream, etc.	<7.35	5	1	1
		7.35-7.94	4	2	2
		7.94-8.69	3	3	3
		8.69-13.98	2	4	4
		>13.98	1	5	5
Animal fat	Lard and butter	<1.96	5	5	5
		1.96-2.02	4	4	4
		2.02-2.09	3	3	3
		2.09-4.14	2	2	2
		>4.14	1	1	1
Dairy	Whole fat milk, low fat milk, powdered milk (whole fat), powdered milk (low fat), yogurt, and cheese	<54.81	5	5	5
		54.81-82.74	4	4	4
		82.74-181.88	3	3	3
		181.88-318.05	2	2	2
		>318.05	1	1	1
Eggs	Fresh egg, duck egg, etc.	<42.23	5	5	5
		42.23-55.37	4	4	4

		55.37-64.97	3	3	3
		64.97-76.96	2	2	2
		>76.96	1	1	1
Fish or seafood	Fresh water fish (silver carp, bream, crucian carp, etc.), salt water fish (yellow croaker, hair tail, etc.), shrimp or crab, conch, rice field eel or river eel, salted fish or processed fishery products	<12.03	5	5	5
		12.03-13.89	4	4	4
		13.89-16.73	3	3	3
		16.73-22.57	2	2	2
		>22.57	1	1	1
Meat	Fresh pork, fresh beef or lamb, poultry (chicken, duck, goose, etc.), animal livers, organ meat (heart, brain, tongue, tripe, intestine, etc.), and processed meat (sausage, spam, etc.)	<18.74	5	5	5
		18.74-27.03	4	4	4
		27.03-42.49	3	3	3
		42.49-65.69	2	2	2
		>65.69	1	1	1
Micellaneous animal based foods	Meat bun and meat pie	<7.52	5	5	5
		7.52-10.94	4	4	4
		10.94-15.46	3	3	3
		15.46-36.24	2	2	2
		>36.24	1	1	1

* Eighteen food groups were assigned either positive or reverse scores after segregation into quintiles. Participants with an intake above the highest quintile for the positive score received a score of 5. Those below the lowest quintile intake received a score of 1. A reverse value was applied for the reverse scores. The scores for each participant were summed to create the final score.

Table S2. The intakes of food groups based on the presence of recurrent oral ulcer.

Food groups	Recurrent oral ulcer		*p*-Value
	Yes (N=337)	No (N=242)	
Refined grains (g/day)	183.75 (93.83)	183.53 (104.45)	0.564
Whole grains (g/day)	58.31 (53.28)	64.89 (45.89)	0.008
Legumes (g/day)	18.82 (17.48)	18.61 (13.69)	0.368
Vegetables (g/day)	368.63 (212.51)	368.48 (129.43)	0.167
Mushroom (g/day)	27.18 (31.65)	26.98 (36.08)	0.677
Fruits (g/day)	193.61 (135.79)	190.52 (136.91)	0.642
Nuts (g/day)	6.61 (7.07)	7.27 (7.50)	0.439
Red meat (g/day)	24.41 (25.80)	20.83 (21.14)	0.023
Processed meat (g/day)	7.65 (10.28)	6.19 (9.81)	<0.001
Poultry (g/day)	11.33 (14.03)	10.00 (17.99)	0.013
Fish (g/day)	19.48 (26.63)	15.24 (9.76)	0.006
Dairy (g/day)	249.47 (205.12)	244.62 (287.62)	<0.001
Eggs (g/day)	63.96 (34.55)	60.79 (32.81)	0.205

Data are presented as median (interquartile ranges). *p*-Values were based on Wilcoxon rank sum test.

Table S3. Daily intake ranges for tertiles of 13 food groups.

Food groups	T1	T2	T3
Refined grains (g/day)	<122.86	122.86-202.00	>202.00
Whole grains (g/day)	<57.14	57.14-85.71	>85.71
Legumes (g/day)	<10.48	10.48-19.34	>19.34
Vegetables (g/day)	<296.47	296.47-414.28	>414.28
Mushroom (g/day)	<10.00	10.00-33.57	>33.57
Fruits (g/day)	<105.59	105.59-240.22	>240.22
Nuts (g/day)	<2.50	2.50-3.57	>3.57
Red meat (g/day)	<8.81	8.81-30.24	>30.24
Processed meat (g/day)	<3.33	3.33-6.67	>6.67
Poultry (g/day)	<5.00	5.00-7.14	>7.14
Fish (g/day)	<9.67	9.67-15.67	>15.67
Dairy (g/day)	<138.99	138.99-253.13	>253.13
Eggs (g/day)	<52.32	52.32-67.65	>67.65

Thirteen food groups were divided by tertiles of daily intakes.

Table S4. The distribution of recurrent oral ulcer among tertile groups of all dietary factors.

Dietary factors	Recurrent oral ulcer		*p*-Value
	Yes (N=337)	No(N=242)	
PDI			0.187
T1	133 (39.5%)	81 (33.5%)	
T2	118 (35.0%)	84 (34.7%)	
T3	86 (25.5%)	77 (31.8%)	
hPDI			<0.001
T1	138 (40.9%)	53 (21.9%)	
T2	120 (35.6%)	91 (37.6%)	
T3	79 (23.4%)	98 (40.5%)	
uPDI			0.840
T1	113 (33.5%)	80 (33.1%)	
T2	115 (34.1%)	88 (36.4%)	
T3	109 (32.3%)	74 (30.6%)	
Refined grains			0.435
T1	118 (35.0%)	96 (39.7%)	
T2	115 (34.1%)	72 (29.8%)	
T3	104 (30.9%)	74 (30.6%)	
Whole grains			0.006
T1	222 (65.9%)	131 (54.1%)	
T2	27 (8.0%)	18 (7.4%)	
T3	88 (26.1%)	93 (38.4%)	
Legumes			0.707
T1	115 (34.1%)	76 (31.4%)	
T2	115 (34.1%)	82 (33.9%)	
T3	107 (31.8%)	84 (34.7%)	
Vegetables			0.283
T1	120 (35.6%)	71 (29.3%)	
T2	112 (33.2%)	87 (36.0%)	
T3	105 (31.2%)	84 (34.7%)	
Mushroom			0.785
T1	131 (38.9%)	101 (41.7%)	
T2	109 (32.3%)	75 (31.0%)	
T3	97 (28.8%)	66 (27.3%)	
Fruits			0.926
T1	109 (32.3%)	82 (33.9%)	
T2	116 (34.4%)	81 (33.5%)	
T3	112 (33.2%)	79 (32.6%)	
Nuts			0.509
T1	156 (46.3%)	109 (45.0%)	
T2	85 (25.2%)	54 (22.3%)	
T3	96 (28.5%)	79 (32.6%)	
Red meat			0.047
T1	112 (33.2%)	99 (40.9%)	
T2	116 (34.4%)	86 (35.5%)	
T3	109 (32.3%)	57 (23.6%)	
Processed meat			0.001
T1	180 (53.4%)	162 (66.9%)	

T2	85 (25.2%)	53 (21.9%)	
T3	72 (21.4%)	27 (11.2%)	
Poultry			0.127
T1	180 (53.4%)	149 (61.6%)	
T2	82 (24.3%)	52 (21.5%)	
T3	75 (22.3%)	41 (16.9%)	
Fish			0.029
T1	117 (34.7%)	108 (44.6%)	
T2	98 (29.1%)	68 (28.1%)	
T3	122 (36.2%)	66 (27.3%)	
Dairy			<0.001
T1	104 (30.9%)	111 (45.9%)	
T2	138 (40.9%)	89 (36.8%)	
T3	95 (28.2%)	42 (17.4%)	
Eggs			0.105
T1	110 (32.6%)	81 (33.5%)	
T2	105 (31.2%)	92 (38.0%)	
T3	122 (36.2%)	69 (28.5%)	

Data are presented as n (percentage) for categorical variables. The percentage sum of some cells is not equal to 100 because the percentage was rounded to retain decimal places. *p*-Values were based on Chi-squared test. *PDI* plant-based diet index, *hPDI* healthful plant-based diet index, *uPDI* unhealthful plant-based diet index.

Table S5. Distributions of chronic systemic diseases of study participants

Diseases	n	Prevalence
Hypertension	51	8.81%
Cardiovascular diseases	50	8.64%
Diabetes	19	3.28%
Tumor	13	2.25%
Gastritis	12	2.07%
Osteoarthritis	11	1.90%
Hepatitis	11	1.90%
Gastroenteritis	11	1.90%
Otorhinolaryngological diseases	10	1.73%
Endocrine system diseases	8	1.38%
Nervous system diseases	8	1.38%
Lumbar disc herniation	7	1.21%
Gynecological diseases	5	0.86%
Hematologic diseases	5	0.86%
Rheumatoid arthritis	5	0.86%
Eye diseases	4	0.69%
Respiratory diseases	4	0.69%
Cancer	4	0.69%
Kidney diseases	3	0.52%
Skin diseases	2	0.35%
Others	3	0.52%

The number of the participants who have a certain chronic systemic disease and the prevalence of this condition has been quantified.

Table S6. Associations of dietary factors with recurrent oral ulcer.

Dietary factors	Odds ratio	95% CI	*p*-Value	*p* for trend
PDI				0.140
T2 vs T1	0.99	0.64-1.54	0.982	
T3 vs T1	0.68	0.42-1.11	0.122	
hPDI				0.043
T2 vs T1	0.82	0.51-1.32	0.404	
T3 vs T1	0.55	0.33-0.92	0.022	
uPDI				0.477
T2 vs T1	0.86	0.55-1.34	0.504	
T3 vs T1	0.85	0.52-1.39	0.523	
Refined grains				0.127
T2 vs T1	1.51	0.97-2.35	0.070	
T3 vs T1	1.53	0.94-2.48	0.085	
Whole grains				0.208
T2 vs T1	0.99	0.50-1.98	0.982	
T3 vs T1	0.75	0.50-1.13	0.172	
Legumes				0.821
T2 vs T1	1.07	0.69-1.67	0.762	
T3 vs T1	0.97	0.60-1.55	0.886	
Vegetables				0.779
T2 vs T1	0.94	0.60-1.48	0.795	
T3 vs T1	0.91	0.56-1.48	0.711	
Mushroom				0.667
T2 vs T1	1.04	0.67-1.61	0.864	
T3 vs T1	0.93	0.57-1.51	0.765	
Fruits				0.773
T2 vs T1	0.92	0.59-1.44	0.716	
T3 vs T1	0.94	0.58-1.53	0.811	
Nuts				0.927
T2 vs T1	1.12	0.71-1.78	0.619	
T3 vs T1	1.00	0.65-1.56	0.986	
Red meat				0.013
T2 vs T1	1.19	0.78-1.84	0.419	
T3 vs T1	1.89	1.14-3.15	0.014	
Processed meat				0.591
T2 vs T1	0.91	0.57-1.44	0.689	
T3 vs T1	1.30	0.73-2.31	0.367	
Poultry				0.342
T2 vs T1	1.10	0.70-1.75	0.678	
T3 vs T1	1.27	0.76-2.11	0.366	
Fish				0.297
T2 vs T1	1.53	0.98-2.39	0.063	
T3 vs T1	1.35	0.86-2.13	0.195	

Dairy				0.555
T2 vs T1	1.26	0.83-1.90	0.281	
T3 vs T1	1.31	0.77-2.22	0.323	
Eggs				0.025
T2 vs T1	1.11	0.71-1.74	0.651	
T3 vs T1	1.74	1.06-2.83	0.028	

Odds ratios were derived from logistic regression models for tertiles 2 (T2) and tertiles 3 (T3) of included dietary factors using tertiles 1 (T1) as the reference group. Co-variates included age, sex, current smoking status, physical activity, total energy intake, BMI, common chronic diseases, brushing and oral conditions. *PDI* plant-based diet index, *hPDI* healthful plant-based diet index, *uPDI* unhealthful plant-based diet index.

Table S7. Associations of genera with ROU after further adjusting for brushing and oral conditions.

Genera	Coefficient	*p*-Value	*p*-FDR
Atopobium	0.049	0.016	0.115
TM7x	0.038	0.005	0.050
Roseburia	-0.007	0.022	0.143
unclassified Muribaculaceae	-0.010	0.001	0.012
Rikenellaceae RC9 gut group	-0.011	0.002	0.018
Gracilibacteria	-0.013	0.003	0.029
Abiotrophia	-0.026	0.023	0.147
Bergeyella	-0.038	0.014	0.105

Coefficient is the effect value obtained from MaAsLin2. *MaAsLin* Multivariate Analysis by Linear Models. *ROU* recurrent oral ulcer. · $p < 0.10$, * $p < 0.05$, ** $p < 0.01$, *** $p < 0.001$.

Table S8. Associations of genera with ROU using MaAsLin2 and ANCOM-BC.

Genus	MaAsLin2			ANCOM-BC	
	Coefficient	*p*-Value	*p*-FDR	Coefficient	*p*-Value
Leptotrichia	0.083	0.057	0.183	0.129	0.021
Actinomyces	0.060	0.056	0.182	0.110	0.023
Atopobium	0.049	0.016	0.085	0.182	0.001
TM7x	0.038	0.005	0.032	0.166	0.009
Corynebacterium	0.034	0.044	0.159	0.121	0.018
Wolinella	-0.005	0.047	0.161	0.027	0.798
Family XIII UCG-001	-0.005	0.047	0.161	0.152	0.028
Roseburia	-0.007	0.022	0.102	-0.167	0.129
Unclassified Muribaculaceae	-0.010	0.001	0.008	-0.483	0.001
Olsenella	-0.011	0.041	0.154	-0.080	0.465
Rikenellaceae RC9 gut group	-0.011	0.002	0.013	-0.015	0.877
Peptococcus	-0.012	0.048	0.161	-0.029	0.450
Streptobacillus	-0.013	0.038	0.147	-0.290	0.026
Gracilibacteria	-0.014	0.002	0.017	-0.194	0.131
Abiotrophia	-0.026	0.022	0.104	-0.173	0.026

Bergeyella	-0.039	0.012	0.067	-0.078	0.172

Associations of genera with ROU were analyzed by MaAsLin2 and ANCOM-BC. Coefficients were obtained from MaAsLin2 and ANCOM-BC. *ROU,* recurrent oral ulcer. *MaAsLin* Microbiome Multivariable Associations with Linear Models. *ANCOM-BC* Analysis of Compositions of Microbiomes with Bias Correction.

Table S9. Associations of genera with ROU in different sexes.

**Genus**	**Male**		**Female**		***p*-interaction**
	**Coefficient**	***p*-Value**	**Coefficient**	***p*-Value**	
Leptotrichia	0.115	0.135	0.060	0.258	0.353
Actinomyces	0.135	0.031*	0.015	0.662	0.104
Atopobium	0.098	0.022*	0.025	0.257	0.245
TM7x	0.029	0.274	0.045	0.005**	0.136
Corynebacterium	0.079	0.003**	0.014	0.514	0.075·
Wolinella	0.001	0.883	-0.008	0.002**	0.452
Family_XIII_UCG.001	-0.002	0.633	-0.006	0.030*	0.867
Roseburia	-0.009	0.229	-0.007	0.016*	0.787
unclassified Muribaculaceae	-0.004	0.499	-0.013	0.000***	0.072·
Olsenella	-0.020	0.091·	-0.006	0.245	0.872
Rikenellaceae RC9 gut group	-0.011	0.090·	-0.011	0.005**	0.458
Peptococcus	-0.019	0.045*	-0.008	0.267	0.961
Streptobacillus	-0.002	0.903	-0.018	0.004**	0.999
Gracilibacteria	-0.004	0.507	-0.018	0.002**	0.431
Abiotrophia	-0.020	0.261	-0.029	0.041*	0.783
Bergeyella	-0.004	0.790	-0.053	0.014*	0.089·

Associations of genera with ROU in different sexes were analyzed by MaAsLin2. Coefficients were the effect values obtained from MaAsLin2. *p*-interaction of sex is obtained from the linear model. *MaAsLin* Multivariate Analysis by Linear Models. *ROU* recurrent oral ulcer. · $p < 0.10$, * $p < 0.05$, ** $p < 0.01$, *** $p < 0.001$.

Table S10 MetaCyc pathways significantly associated with ROU as identified by MaAsLin2.

Pathway	Description	Ontology-pathway type	Coefficient	*p*-Value	*p*-FDR
PWY0-1297	superpathway of purine deoxyribonucleosides degradation	Nucleoside and Nucleotide Degradation	0.009	0.022	0.065
P161-PWY	acetylene degradation (anaerobic)	Generation of Precursor Metabolites and Energy	0.008	0.029	0.078
LACTOSECAT-PWY	lactose degradation I	Carbohydrate Degradation	0.008	0.042	0.102
PWY-5505	L-glutamate and L-glutamine biosynthesis	Amino Acid Biosynthesis	0.008	0.018	0.056
PWY-621	sucrose degradation III (sucrose invertase)	Carbohydrate Degradation	0.007	0.064	0.139
PWY0-1298	superpathway of pyrimidine deoxyribonucleosides degradation	Nucleoside and Nucleotide Degradation	0.007	0.071	0.15
PWY-5910	superpathway of geranylgeranyldiphosphate biosynthesis I (via mevalonate)	Secondary Metabolite Biosynthesis	0.007	0.063	0.137
PWY-7392	taxadiene biosynthesis (engineered)	Secondary Metabolite Biosynthesis	0.007	0.081	0.166
PWY-6470	peptidoglycan biosynthesis V (β-lactam resistance)	Antibiotic Resistance	0.006	0.055	0.124
P124-PWY	Bifidobacterium shunt	Generation of Precursor Metabolites and Energy	0.005	0.014	0.048
P122-PWY	heterolactic fermentation	Generation of Precursor Metabolites and Energy	0.005	0.006	0.024
P441-PWY	superpathway of N-acetylneuraminate degradation	Carboxylic Acid Degradation	0.005	0.026	0.074
PWY-7003	glycerol degradation to butanol	Generation of Precursor Metabolites and Energy	0.004	0.046	0.108
PWY-6353	purine nucleotides degradation II (aerobic)	Nucleoside and Nucleotide Degradation	0.004	0.058	0.13
PWY-4984	urea cycle	Inorganic Nutrient Metabolism	0.004	0.049	0.114
P4-PWY	superpathway of L-lysine, L-threonine and L-methionine biosynthesis I	Amino Acid Biosynthesis	0.004	0.01	0.038

P164-PWY	purine nucleobases degradation I (anaerobic)	Generation of Precursor Metabolites and Energy	0.004	0.007	0.029
SALVADEHYPOX-PWY	adenosine nucleotides degradation II	Nucleoside and Nucleotide Degradation	0.003	0.036	0.091
UDPNAGSYN-PWY	UDP-N-acetyl-D-glucosamine biosynthesis I	Carbohydrate Biosynthesis	0.003	0.055	0.124
PWY-6478	GDP-D-glycero-α-D-manno-heptose biosynthesis	Carbohydrate Biosynthesis	0.003	0.046	0.108
PWY-7187	pyrimidine deoxyribonucleotides de novo biosynthesis II	Nucleoside and Nucleotide Biosynthesis	0.003	0.021	0.062
PWY-6608	guanosine nucleotides degradation III	Nucleoside and Nucleotide Degradation	0.003	0.062	0.136
GLUCUROCAT-PWY	superpathway of β-D-glucuronosides degradation	Carbohydrate Degradation	0.003	0.06	0.134
PWY-5265	peptidoglycan biosynthesis II (staphylococci)	Cell Structure Biosynthesis	0.003	0.017	0.054
PWY0-781	aspartate superpathway	Superpathways	0.002	0.076	0.158
P562-PWY	myo-inositol degradation I	Cyclitol Degradation	0.002	0.063	0.138
PWY-5973	cis-vaccenate biosynthesis	Fatty Acid and Lipid Biosynthesis	-0.003	0.087	0.177
RIBOSYN2-PWY	flavin biosynthesis I (bacteria and plants)	Cofactor, Carrier, and Vitamin Biosynthesis	-0.003	0.099	0.195
PWY-7539	6-hydroxymethyl-dihydropterin diphosphate biosynthesis III (Chlamydia)	Other Biosynthesis	-0.005	0.005	0.022
PANTO-PWY	phosphopantothenate biosynthesis I	Cofactor, Carrier, and Vitamin Biosynthesis	-0.005	0.07	0.149
PWY0-1319	CDP-diacylglycerol biosynthesis II	Fatty Acid and Lipid Biosynthesis	-0.005	0.032	0.083
ASPASN-PWY	superpathway of L-aspartate and L-asparagine biosynthesis	Amino Acid Biosynthesis	-0.005	0.042	0.102
PWY-7560	methylerythritol phosphate pathway II	Secondary Metabolite Biosynthesis	-0.005	0.025	0.072
PWY-6467	Kdo transfer to lipid IVA (Chlamydia)	Cell Structure Biosynthesis	-0.005	0.046	0.108

PWY-6897	thiamine diphosphate salvage II	Cofactor, Carrier, and Vitamin Biosynthesis	-0.005	0.034	0.087
THISYN-PWY	superpathway of thiamine diphosphate biosynthesis I	Cofactor, Carrier, and Vitamin Biosynthesis	-0.006	0.026	0.074
PWY-6703	preQ0 biosynthesis	Secondary Metabolite Biosynthesis	-0.006	0.085	0.174
PWY-6700	queuosine biosynthesis I (de novo)	Nucleic Acid Processing	-0.006	0.018	0.056
FASYN-ELONG-PWY	fatty acid elongation -- saturated	Fatty Acid and Lipid Biosynthesis	-0.006	0.004	0.018
PYRIDNUCSYN-PWY	NAD de novo biosynthesis I	Cofactor, Carrier, and Vitamin Biosynthesis	-0.006	0.033	0.085
PWY-1269	CMP-3-deoxy-D-manno-octulosonate biosynthesis	Carbohydrate Biosynthesis	-0.007	0.021	0.063
NAGLIPASYN-PWY	lipid IVA biosynthesis (E. coli)	Cell Structure Biosynthesis	-0.008	0.017	0.053
PYRIDOXSYN-PWY	pyridoxal 5'-phosphate biosynthesis I	Cofactor, Carrier, and Vitamin Biosynthesis	-0.008	0.064	0.139
PWY-6519	8-amino-7-oxononanoate biosynthesis I	Other Biosynthesis	-0.01	0.062	0.137
PPGPPMET-PWY	ppGpp metabolism	Metabolic Regulator Biosynthesis	-0.011	0.094	0.186

ROU, recurrent oral ulcer; *MaAsLin*, multivariate analysis by linear models. *FDR*, false discovery rate. Only results with FDR $q < 0.20$ were displayed in this table.

Table S11. Associations of ROU-associated genera with hPDI in different sexes.

Genus	*p*-Value		*p*-interaction
	Male	Female	
Leptotrichia	0.362	0.247	0.209
Actinomyces	0.877	0.645	0.880
Atopobium	0.570	0.024*	0.556
TM7x	0.783	0.002**	0.126
Corynebacterium	0.654	0.010*	0.211
Wolinella	0.747	0.721	0.708
Family XIII UCG-001	0.081·	0.005**	0.579
Roseburia	0.001**	0.247	0.057·
unclassified Muribaculaceae	0.015*	0.001**	0.585
Olsenella	0.039*	0.000***	0.808
Rikenellaceae RC9 gut group	0.228	0.030*	0.008**
Peptococcus	0.134	0.477	0.385
Streptobacillus	0.389	0.072·	0.074·
Gracilibacteria	0.952	0.643	0.650
Abiotrophia	0.747	0.001**	0.082·
Bergeyella	0.056·	0.013*	0.839

Associations of the ROU-associated genera with hPDI in different sexes were analyzed by Wilcoxon rank sum test. *p*-interaction of sex is obtained from the linear model. *ROU* recurrent oral ulcer. *hPDI* healthful plant-based diet index. · $p < 0.10$, * $p < 0.05$, ** $p < 0.01$, *** $p < 0.001$.

Table S12. Associations of ROU-associated genera with red meat intakes in different sexes.

Genus	*p*-Value		*p*-interaction
	Male	Female	
Leptotrichia	0.769	0.603	0.699
Actinomyces	0.564	0.520	0.381
Atopobium	0.852	0.882	0.967
TM7x	0.463	0.235	0.162
Corynebacterium	0.513	0.582	0.529
Wolinella	0.089·	0.174	0.724
Family_XIII_UCG.001	0.984	0.368	0.620
Roseburia	0.157	0.122	0.264
unclassified Muribaculaceae	0.023*	0.527	0.182
Olsenella	0.000***	0.064·	0.120
Rikenellaceae RC9 gut group	0.870	0.396	0.820
Peptococcus	0.046*	0.573	0.086·
Streptobacillus	0.317	0.001**	0.299
Gracilibacteria	0.609	0.455	0.754
Abiotrophia	0.255	0.108	0.346
Bergeyella	0.010*	0.888	0.016*

Associations of the ROU-associated genera with red meat intakes in different sexes were analyzed by Wilcoxon rank sum test. *p*-interaction of sex is obtained from the linear model. · $p < 0.10$, * $p < 0.05$, ** $p < 0.01$, *** $p < 0.001$.

Table S13. Associations of ROU-associated genera with egg intakes in different sexes.

Genus	*p*-Value		*p*-interaction
	Male	Female	
Leptotrichia	0.471	0.136	0.838
Actinomyces	0.008**	0.322	0.002**
Atopobium	0.011*	0.286	0.017*
TM7x	0.072	0.806	0.845
Corynebacterium	0.318	0.866	0.343
Wolinella	0.702	0.990	0.490
Family_XIII_UCG.001	0.402	0.458	0.812
Roseburia	0.282	0.119	0.513
unclassified Muribaculaceae	0.277	0.239	0.687
Olsenella	0.136	0.436	0.532
Rikenellaceae RC9 gut group	0.231	0.010*	0.395
Peptococcus	0.863	0.927	0.237
Streptobacillus	0.373	0.573	0.034*
Gracilibacteria	0.811	0.205	0.243
Abiotrophia	0.579	0.163	0.711
Bergeyella	0.676	0.445	0.141

Associations of the ROU-associated genera with egg intakes in different sexes were analyzed by Wilcoxon rank sum test. *p*-interaction of sex is obtained from the linear model. * $p < 0.05$, ** $p < 0.01$.
